# High prevalence and distinct patterns of metabolic syndrome in rheumatoid arthritis, psoriatic arthritis, and axial spondyloarthritis: a population-based study

**DOI:** 10.1007/s00296-025-05970-9

**Published:** 2025-09-25

**Authors:** Jacob Corum Williams, Kira Rogers, Joshua Southworth, Ryan Malcolm Hum, Pauline Ho, Sizheng Steven Zhao

**Affiliations:** 1 St James’s University Hospital, Leeds Teaching Hospitals NHS Trust, Leeds, UK; 2 Leeds Institute of Rheumatic and Musculoskeletal Medicine, University of Leeds, Leeds, UK; 3 School of Medical Sciences, University of Manchester, Manchester, UK; 4 Versus Arthritis Centre for Genetics and Genomics, Centre for Musculoskeletal Research, The University of Manchester, Manchester, UK; 5 NIHR Manchester Biomedical Research Centre, Manchester University NHS Foundation Trust, Manchester Academic Health Science Centre, Manchester, UK; 6 The Kellgren Centre for Rheumatology, Manchester Royal Infirmary, Manchester, UK; 7 Centre for Musculoskeletal Research , The University of Manchester , Manchester, UK

**Keywords:** Arthritis, Rheumatoid, Arthritis, Psoriatic, Spondylitis, Ankylosing, Obesity, Metabolic Syndrome, Epidemiology

## Abstract

Introduction: Metabolic syndrome (MetS) in inflammatory arthritis (IA) directly impacts its management and associated morbidity and mortality. MetS is a well-recognised comorbidity in PsA, but the epidemiology across IA is unclear. This study aimed to characterise the prevalence of MetS across rheumatoid arthritis (RA), psoriatic arthritis (PsA) and axial spondyloarthritis (axSpA) compared to controls. Methods: We performed a cross-sectional analysis of half a million individuals from the UK Biobank, aged 40 to 69 years, who were collected between 2006 and 2010. Participants with RA, PsA, and axSpA were identified using ICD-10 codes and/or read codes. MetS was defined according to adapted National Cholesterol Education Program Adult Treatment Panel III criteria. Statistical analysis included ANOVA and chi-squared test for between-group difference and logistic regression for odds of MetS, adjusted for age, sex, CRP and smoking status. Results: The prevalence of MetS was highest in RA (43.4%), followed by PsA (42.3%), axSpA (37.1%) and controls (31.8%). Hypertension was prevalent across all IAs (~ 80%), as was hypertriglyceridaemia. Elevated waist circumference and dysglycaemia were more prevalent in RA and PsA compared to axSpA. The adjusted odds of comorbid MetS were elevated in RA (OR 1.15; 95% CI 1.07, 1.24; *p* < 0.001) and PsA (OR 1.31; 95% CI 1.13, 1.52; *p* < 0.001) compared to controls, but decreased in axSpA (OR 0.82; 95% CI 0.70, 0.96; *p* = 0.012). Conclusion: RA and PsA, but not axSpA, are associated with an increased odds of MetS. Holistic management strategies that address both IA and MetS are essential for improving mortality and morbidity.

**Supplementary Information:**

The online version contains supplementary material available at 10.1007/s00296-025-05970-9.

## Introduction

Inflammatory arthritis (IA), including rheumatoid arthritis (RA), psoriatic arthritis (PsA), and axial spondyloarthritis (axSpA), share common immunogenetic mechanisms, clinical features, and therapeutic options [1]. These conditions also have a potentially bidirectional relationship with metabolic syndrome (MetS); for example, obesity is a shared risk factor, and systemic inflammation in IA may contribute to MetS features like insulin resistance [2, 3]. This association has clinical implications; for instance, metabolic dysfunction-associated steatotic liver disease (MASLD) can restrict the use of liver-toxic disease-modifying antirheumatic drugs (DMARDs) [4], while obesity may affect treatment response through immune modulation and/or pharmacodynamics [5]. 

MetS affects an estimated quarter of the general population, with prevalence expected to rise in light of the global obesity epidemic [6]. It is characterised by central adiposity, high blood pressure, and abnormalities in serum glucose and lipid levels [7], and often co-exists with obesity [8]. MetS is commonly defined using criteria from the 2001 National Cholesterol Education Program (NCEP) Adult Treatment Panel III (ATP-III) or the 2009 International Diabetes Federation (IDF) Task Force Joint Interim Statement [7, 9]. 

The prevalence of MetS is higher among individuals with IA compared to the general population. The prevalence of MetS has been reported as 31% in RA and 46% in PsA, with the prevalence in axSpA estimated to be around 33% [10, 11]. In contrast, the prevalence in the general population is 25.4% [6]. However, these studies are highly heterogeneous in terms of geographic location, IA classification and MetS definition, making comparisons between types of IA challenging [10]. Furthermore, few studies have directly compared MetS prevalence across RA, PsA, and axSpA. Better understanding the epidemiology of MetS across IA is important because it directly influences treatment response, medication choice and the risks of cardiovascular disease (CVD), type 2 diabetes, and all-cause mortality [4, 7, 8, 12, 13]. Understanding the relative risk of MetS between different IAs is essential to providing holistic care to the groups most at risk. We aimed to describe the prevalence of MetS in a large cohort of individuals with RA, PsA, or axSpA.

## Methods

We conducted a cross-sectional study using data from the UK Biobank, a cohort study of over 500,000 individuals aged 40 to 69 years recruited between 2006 and 2010. Ethical approval for the study was obtained, and details are available elsewhere [14]. Cross-sectional data from the baseline assessment, performed between 2006 and 2010, were used for the current observational analysis.

Participants with RA, PsA, and axSpA were identified through (1) ICD-10 codes from hospital admission records and/or (2) read codes from primary care records. Individuals with more than one code, for whom the precise diagnosis cannot be ascertained, were excluded. Controls were individuals without a coded diagnosis of RA, PsA or axSpA. Controls with a self-reported diagnosis of RA, PsA or axSpA were also excluded.

MetS was defined using a modified version of the NCEP ATP III criteria [9] (not using fasting blood samples), requiring at least three of the following five criteria: (1) a waist circumference of ≥ 102 cm (40 inches) in men or ≥ 88 cm (35 inches) in women; (2) blood pressure > 130/85 mmHg, a recorded diagnosis of hypertension, or use of antihypertensive medication; (3) a random glucose level ≥ 5.6 mmol/L (≥ 100 mg/dL), a diagnosis of type 2 diabetes, or use of diabetes medications (excluding insulin to avoid misclassification of type 1 diabetes); (4) a random triglyceride level ≥ 1.693 mmol/L (≥ 150 mg/dL); and (5) an HDL cholesterol level < 1.0 mmol/L (< 40 mg/dL) in men or < 1.293 mmol/L (< 50 mg/dL) in women, or use of lipid-lowering therapy.

We used descriptive statistics, presenting means for continuous variables and percentages for categorical variables. Statistical comparison was initially omitted, as the large sample size would likely result in statistically significant differences across all variables, regardless of clinical significance. However, such comparisons were later added at the request of the peer reviewers. For this, continuous variables were compared using ANOVA and categorical using chi-squared test. Prevalence of MetS was compared using a logistic regression model with MetS as the dependent variable and inflammatory arthritis as the independent variable, adjusted for age, sex, CRP and smoking status. Analysis was performed using Stata v15.

## Results

The analysis included 498,961 participants, including 3,220 individuals with RA, 804 with PsA and 827 with axSpA. The remaining 494,110 participants without an IA diagnosis served as controls. The RA group were older (mean age 59.8 years), more likely to be female (71.2%) and had a higher mean CRP (6.5 mg/L, SD 9.3) compared to controls and other IAs.

The prevalence of MetS was higher in all IA groups compared to controls (31.8%), with rates of 43.4% in RA, 42.3% in PsA, and 37.1% in axSpA. Detailed results are presented in Table 1. Notably, the proportion of participants meeting the high blood pressure criteria was similar across IA groups at approximately 80%. Dysglycaemia was most prevalent in RA (21.7%), followed by PsA (20.9%). Similarly, an elevated waist circumference was more common in the RA and PsA groups (45.2% and 44.0%, respectively), but the AxSpA group (33.3%) was similar to the controls (33.6%). Derangement of HDLs was most common in RA (44.5%), whereas attainment of the triglyceride criterion was highest in PsA (48.2%). Significant differences were detected between groups across all demographic and metabolic variables.

**Fig. 1:**
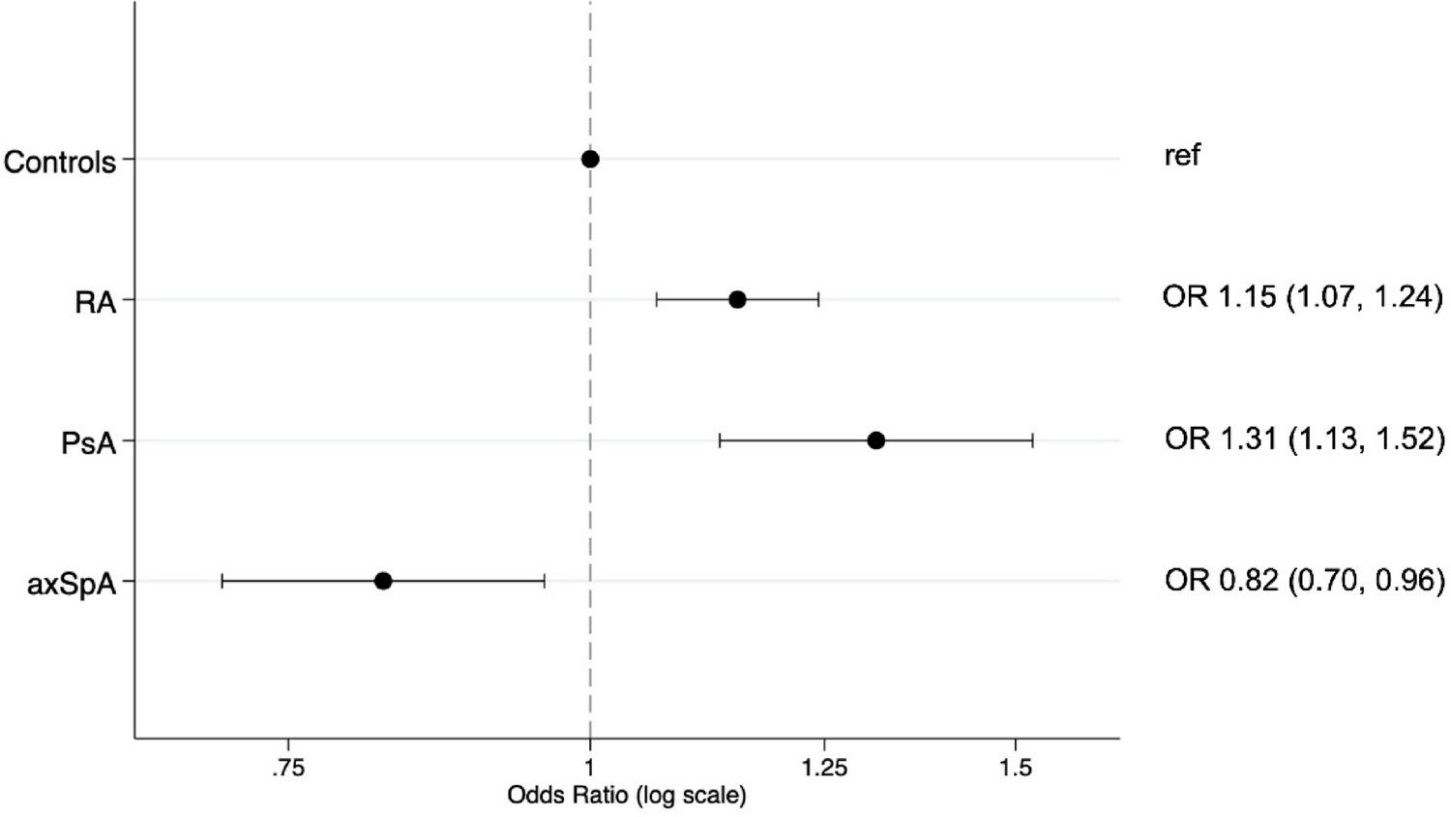
Logistic regression of metabolic syndrome and inflammatory arthritis, adjusted for age, sex, CRP, and smoking history. Definitions: axSpA = axial spondyloarthritis; PsA = psoriatic arthritis; RA = rheumatoid arthritis

Logistic regression analysis demonstrated increased odds of MetS in RA (OR 1.15; 95% CI 1.07, 1.24; *p* < 0.001) and PsA (OR 1.31; 95% CI 1.13, 1.52; *p* < 0.001) but decreased odds of MetS in axSpA (OR 0.82; 95% CI 0.70, 0.96; *p* = 0.012) compared to controls, after adjusting for age, sex, CRP and smoking status (Fig. 1).

**Table 1:** Baseline demographics and prevalence of MetS and its components

	Controls	RA	PsA	axSpA
n (%)	494,110 (99.0)	3,220 (0.6)	804 (0.2)	827 (0.2)
Mean age at recruitment (SD)	56.5 (8.1)	59.8 (6.9)	56.9 (7.4)	57.6 (7.5)
Male, n (%)	225,731 (45.7%)	927 (28.8%)	421 (52.4%)	610 (73.8%)
CRP (mg/L), mean (SD)	2.6 (4.3)	6.5 (9.3)	5.0 (7.7)	6.1 (8.2)
Body mass index (BMI), mean (SD)	27.4 (4.8)	28.2 (5.5)	28.8 (5.3)	27.4 (4.6)
Current smoker, n (%)	51,937 (10.5%)	390 (12.1%)	72 (9.0%)	98 (11.9%)
Previous smoker, n (%)	169,676 (34.4%)	1379 (42.9%)	331 (41.2%)	354 (42.9%)
Never smoker, n (%)	269,615 (54.7%)	1421 (44.2%)	395 (49.2%)	372 (45.0%)
**Metabolic syndrome, n (%)**	156,648 (31.8%)	1395 (43.4%)	340 (42.3%)	306 (37.1%)
**Fulfilling waist circumference criteria, n (%)**	165,351 (33.6%)	1447 (45.2%)	353 (44.0%)	274 (33.3%)
**Fulfilling high BP criteria, n (%)**	362,086 (73.3%)	2596 (80.6%)	639 (79.5%)	660 (79.8%)
SBP > 130 mmHg or DBP > 85 mmHg, n (%)	334,082 (67.6%)	2280 (70.8%)	574 (71.4%)	585 (70.7%)
On antihypertensives, n (%)	108,693 (38.7%)	1206 (68.8%)	264 (49.3%)	263 (40.0%)
Previous diagnosis of HTN, n (%)	132,783 (27.0%)	1199 (37.4%)	308 (38.5%)	288 (35.0%)
**Fulfilling glucose criteria, n (%)**	74,925 (15.2%)	697 (21.7%)	168 (20.9%)	134 (16.2%)
Baseline glucose ≥ 5.6 mmol/L, n (%)	63,587 (15.1%)	556 (20.5%)	145 (20.7%)	110 (15.4%)
Previous diagnosis of T2DM, n (%)	25,726 (5.2%)	311 (9.7%)	56 (7.0%)	54 (6.5%)
On antidiabetic medication, n (%)	18,523 (3.7%)	223 (6.9%)	47 (5.8%)	37 (4.5%)
**Fulfilling triglycerides criteria, n (%)**	201,429 (41.9%)	1419 (44.8%)	379 (48.2%)	359 (45.2%)
**Fulfilling cholesterol criteria, n (%)**	146,512 (31.3%)	1385 (44.5%)	293 (38.9%)	287 (37.7%)
HDL cholesterol < 1 mmol/L (m) or < 1.293 mmol/L (w), n (%)	83,620 (19.8%)	767 (28.3%)	181 (25.8%)	144 (20.2%)
On cholesterol-lowering medication, n (%)	84,543 (27.4%)	896 (34.4%)	160 (33.7%)	188 (50.8%)

*p* < 0.001 for all variables compared using ANOVA for continuous and chi-squared for categorical variables. Rows highlighted in **bold** reflect the diagnostic criteria of metabolic syndrome.Waist circumference criteria: waist measurement ≥ 102 cm (40 inches) in men OR ≥ 88 cm (35 inches) in women; BP criteria: baseline visit blood pressure measurements > 130/85 mmHg OR Diagnosis of hypertension OR Use of antihypertensives; Glucose criteria: baseline visit random glucose measurements ≥ 5.6 mmol/L (≥ 100 mg/dl) OR diagnosis of type 2 diabetes mellitus OR use of diabetes medications; Triglyceride criteria: baseline visit random triglyceride levels ≥ 1.693 mmol/l (> 150 mg/dL); Cholesterol criteria: baseline random HDL levels < 1 mmol/l (< 40 mg/dL) in men or < 1.293 mmol/l (< 50 mg/dL) in women OR Use of lipid-lowering mediation.Definitions. axSpA = axial spondyloarthritis, BP = blood pressure, CRP = C-reactive protein, DBP = diastolic blood pressure, HDL = high density lipoprotein, HTN = hypertension, m = men, MetS = metabolic syndrome, n = number, PsA = psoriatic arthritis, SBP = systolic blood pressure, RA = rheumatoid arthritis, SD = standard deviation, T2DM = type 2 diabetes mellitus, w = women

## Discussion

By directly comparing the prevalence of MetS and its components across RA, PsA, axSpA and controls, this study revealed several notable findings. Firstly, despite MetS being classically associated with PsA [15], RA had a comparable prevalence. Likewise, the odds of comorbid MetS were elevated for both RA and PsA. In contrast, the prevalence of MetS in axSpA was lower than in other IAs, and there were decreased odds of comorbid MetS compared to controls when adjusting for confounders. The prevalence of hypertension and deranged triglycerides and cholesterol was similar in axSpA compared to other IAs, but the prevalence of an elevated waist circumference and dysglycaemia was comparable to controls, resulting in a lower prevalence of MetS overall. Our results also highlight the general prevalence of MetS, with almost a third of our control group meeting the criteria.

Previous studies have reported variable MetS prevalence in IA populations. A large meta-analysis by Hallajzadeh et al. estimated the prevalence of MetS in RA to range from 14.32 to 37.83%, with an overall pooled prevalence of 30.65% [16]. Estimates of the prevalence of MetS in PsA vary, with meta-analyses reporting a range of 29–46% [10, 17]. Data on MetS prevalence in axSpA are limited, with studies estimating it to be between 14% and 34.9% [11, 18, 19]. In a recent cross-sectional study by Guła et al. comparing comorbidities between RA, PsA and axSpA, the axSpA group showed the lowest prevalence of hypertension, obesity, dyslipidaemia and diabetes mellitus, supporting our findings of a lower burden of metabolic comorbidities in these patients compared to other IAs [20]. In the general population, the prevalence of MetS based on the ATP-III definition has been reported as 25.4% globally and 25.3% in Europe [6]. Notably, in our study, the prevalence of MetS and central obesity was similar in RA and PsA.

Although more commonly associated with PsA, MetS is a well-recognised consequence of RA, secondary to chronic inflammation, comorbidities, lifestyle factors and medications [21]. Our findings suggest that MetS prevalence in RA may approximate that of PsA outside of specialist rheumatology services. As disease severity is closely linked to MetS in PsA, these patients may be over-represented in tertiary services compared to other settings [22]. Conversely, the prevalence of MetS may differ with disease duration, particularly as obesity often precedes the diagnosis of PsA [2]. 

Our estimates for MetS in RA and axSpA are higher than those reported in previous studies. Notably, although the odds ratio for PsA was higher when compared to RA, this is due to a lack of precision and should not be over-interpreted. Comparisons across studies are limited due to variations in geographical location, disease classification criteria and MetS definition. The prevalence of MetS within the same population can vary substantially based on the criteria for MetS used. Furthermore, the performance of specific criteria varies by the ethnicity of the study population [23]. Our study is novel as it employed a consistent definition of MetS within the same population, encompassing participants with different IAs, enabling a direct comparison of the prevalence and odds of MetS by IA type.

Multiple mechanisms may explain the strong association between IA and MetS. Both obesity and MetS are characterised by chronic low-grade inflammation driven by adipokine release from visceral fat [8, 10]. Systemic inflammation, secondary to inflammatory joint disorders, is known to promote insulin resistance, atherosclerosis and endothelial dysfunction [3, 10]. In addition, the pharmacological therapies used for IA can impact MetS. For instance, corticosteroids are associated with weight gain [24], leflunomide is linked to hypertension [25] and Janus kinase inhibitors have been associated with dyslipidaemia [26, 27]. Furthermore, participation in exercise among IA patients is often limited due to both disease-related and psychosocial barriers [28]. 

MetS in IA is associated with both increased risk of CVD [8, 10, 11] and treatment non-response [13, 22]. Identifying MetS in IA, particularly RA and PsA, is critical to ensure the safe prescribing of DMARDs and provides an opportunity to offer lifestyle interventions or pharmacological treatments (for example, antihypertensives). In particular, weight loss in RA [29] and PsA [22, 30] has shown benefit on disease activity and with the advent of GLP-1 agonists in obesity management, there may be utility for these drugs as an adjunctive therapy in the future [15]. 

Our study’s strengths include using a large prospective data set of nearly half a million individuals recruited from the general population. Recruiting from the general population, rather than exclusively from specialist centres, enhances the translatability of our results to individuals presenting to primary and secondary care. Importantly, this is one of the first studies directly comparing the prevalence of MetS in RA, PsA and axSpA using the same study population and diagnostic criteria, enabling direct comparisons between these groups.

This study has several limitations. First, the UK Biobank population tends to be healthier and more affluent than the general population. Nonetheless, we observed a high prevalence of MetS within this cohort, suggesting the current literature may be underestimating the scale of this disease in the broader rheumatology and general populations. Second, there is a risk of disease misclassification when using coded or self-reported data, although gender distribution is generally comparable to that reported for these diseases. Although we were able to adjust for several relevant confounders, we were unable to adjust for disease activity and treatment history, which may independently impact the risk of MetS. Lastly, the UK Biobank study did not collect fasting blood samples, which may increase the proportion of participants meeting MetS criteria based on random blood results.

In conclusion, this study provides valuable insights into MetS in IA by directly comparing across RA, PsA, and axSpA. The prevalence and odds of MetS were elevated in those with RA and PsA, but decreased in axSpA. Future research should focus on optimal strategies for managing both MetS and IA as part of a comprehensive treatment approach.

## Supplementary Information

Below is the link to the electronic supplementary material.

Supplementary Material 1

## Data Availability

The UK Biobank dataset used in this paper is available via application directly to the UK Biobank. Applications are assessed for meeting the required criteria for access, including legal and ethics standards. More information regarding data access can be found at www.ukbiobank.ac.uk.

## References

[1] Poudel P, Goyal A, Lappin SL Inflammatory Arthritis. StatPearls [Internet]. Treasure Island (FL): StatPearls Publishing; 2024 [cited 2024 Sep 18]. Available from: http://www.ncbi.nlm.nih.gov/books/NBK507704/

[2] Love TJ, Zhu Y, Zhang Y, Wall-Burns L, Ogdie A, Gelfand JM et al (2012) Obesity and the risk of psoriatic arthritis: a population-based study. Ann Rheum Dis 71:1273–1277 22586165 10.1136/annrheumdis-2012-201299 PMC3645859

[3] Nicolau J, Lequerré T, Bacquet H, Vittecoq O (2017) Rheumatoid arthritis, insulin resistance, and diabetes. Joint Bone Spine 84:411–416 27777170 10.1016/j.jbspin.2016.09.001

[4] Rinella ME, Neuschwander-Tetri BA, Siddiqui MS, Abdelmalek MF, Caldwell S, Barb D et al (2023) AASLD practice guidance on the clinical assessment and management of nonalcoholic fatty liver disease. Hepatology 77:1797 36727674 10.1097/HEP.0000000000000323 PMC10735173

[5] Bapat SP, Whitty C, Mowery CT, Liang Y, Yoo A, Jiang Z et al (2022) Obesity alters pathology and treatment response in inflammatory disease. Nature 604:337 35355021 10.1038/s41586-022-04536-0 PMC9165753

[6] Noubiap JJ, Nansseu JR, Lontchi-Yimagou E, Nkeck JR, Nyaga UF, Ngouo AT et al (2022) Geographic distribution of metabolic syndrome and its components in the general adult population: a meta-analysis of global data from 28 million individuals. Diabetes Res Clin Pract 188:109924 35584716 10.1016/j.diabres.2022.109924

[7] Alberti KGMM, Eckel RH, Grundy SM, Zimmet PZ, Cleeman JI, Donato KA et al (2009) Harmonizing Metabolic Syndrome Circulation 120:1640–1645 19805654 10.1161/CIRCULATIONAHA.109.192644

[8] González-Muniesa P, Mártinez-González M-A, Hu FB, Després J-P, Matsuzawa Y, Loos RJF et al (2017) Obes Nat Rev Dis Primer 3:1–18 10.1038/nrdp.2017.34 28617414

[9] Third Report of the National Cholesterol Education Program (NCEP) Expert Panel on Detection, Evaluation, and Treatment of High Blood Cholesterol in Adults (Adult Treatment Panel III) Final Report | Circulation [Internet]. [cited 2024 Aug 12]. Available from: https://www.ahajournals.org/doi/10.1161/circ.106.25.3143 12485966

[10] Loganathan A, Kamalaraj N, El-Haddad C, Pile K (2021) Systematic review and meta-analysis on prevalence of metabolic syndrome in psoriatic arthritis, rheumatoid arthritis and psoriasis. Int J Rheum Dis 24:1112–1120 34076348 10.1111/1756-185X.14147

[11] Hintenberger R, Affenzeller B, Vladychuk V, Pieringer H (2023) Cardiovascular risk in axial spondyloarthritis—a systematic review. Clin Rheumatol 42:2621–2633 37418034 10.1007/s10067-023-06655-z PMC10497445

[12] Hui WS, Liu Z, Ho SC (2010) Metabolic syndrome and all-cause mortality: a meta-analysis of prospective cohort studies. Eur J Epidemiol 25:375–384 20425137 10.1007/s10654-010-9459-z

[13] Di Minno MND, Peluso R, Iervolino S, Russolillo A, Lupoli R, Scarpa R et al (2014) Weight loss and achievement of minimal disease activity in patients with psoriatic arthritis starting treatment with tumour necrosis factor α blockers. Ann Rheum Dis 73:1157–1162 23771989 10.1136/annrheumdis-2012-202812 PMC4033114

[14] Sudlow C, Gallacher J, Allen N, Beral V, Burton P, Danesh J et al (2015) UK Biobank: An Open Access Resource for Identifying the Causes of a Wide Range of Complex Diseases of Middle and Old Age. PLoS Med [Internet]. [cited 2024 Sep 8];12. Available from: https://www.ncbi.nlm.nih.gov/pmc/articles/PMC4380465/10.1371/journal.pmed.1001779 PMC4380465 25826379

[15] Williams JC, Hum RM, Rogers K, Maglio C, Alam U, Zhao SS (2024) Metabolic syndrome and psoriatic arthritis: the role of weight loss as a disease-modifying therapy. Ther Adv Musculoskelet Dis 16:1759720X241271886 39161788 10.1177/1759720X241271886 PMC11331474

[16] Hallajzadeh J, Safiri S, Mansournia MA, Khoramdad M, Izadi N, Almasi-Hashiani A et al (2017) Metabolic syndrome and its components among rheumatoid arthritis patients: a comprehensive updated systematic review and meta-analysis. PLoS One 12:e0170361 28333949 10.1371/journal.pone.0170361 PMC5363810

[17] Gupta S, Syrimi Z, Hughes DM, Zhao SS (2021) Comorbidities in psoriatic arthritis: a systematic review and meta-analysis. Rheumatol Int 41:275–284 33423070 10.1007/s00296-020-04775-2 PMC7835184

[18] Papadakis JA, Sidiropoulos PI, Karvounaris SA, Vrentzos GE, Spanakis EK, Ganotakis ES et al (2009) High prevalence of metabolic syndrome and cardiovascular risk factors in men with ankylosing spondylitis on anti-TNFalpha treatment: correlation with disease activity. Clin Exp Rheumatol 27:292–298 19473571

[19] Nemes D, Amaricai E, Catan L, Dragoi M, Popa D, Puenea G et al (2013) AB0531 prevalence and differences of the metabolic syndrome in patients with psoriatic arthritis and ankylosing spondylitis. Ann Rheum Dis 72:A951–A951

[20] Guła Z, Łosińska K, Kuszmiersz P, Strach M, Nowakowski J, Biedroń G et al (2024) A comparison of comorbidities and their risk factors prevalence across rheumatoid arthritis, psoriatic arthritis and axial spondyloarthritis with focus on cardiovascular diseases: data from a single center real-world cohort. Rheumatol Int 44:2817–2828 39527279 10.1007/s00296-024-05740-z PMC11618134

[21] Kerekes G, Nurmohamed MT, González-Gay MA, Seres I, Paragh G, Kardos Z et al (2014) Rheumatoid arthritis and metabolic syndrome. Nat Rev Rheumatol 10:691–696 25090948 10.1038/nrrheum.2014.121

[22] Klingberg E, Bilberg A, Björkman S, Hedberg M, Jacobsson L, Forsblad-d’Elia H et al (2019) Weight loss improves disease activity in patients with psoriatic arthritis and obesity: an interventional study. Arthritis Res Ther 21:17 30635024 10.1186/s13075-019-1810-5 PMC6330463

[23] Asato CBH, Nelson-Hurwitz DC, Lee T, Grandinetti A (2021) Comparative analysis of metabolic syndrome diagnostic criteria and its effects on prevalence in a multiethnic population. Metab Syndr Relat Disord 19:347–351 33650889 10.1089/met.2020.0090 PMC8380796

[24] Baker JF, Sauer BC, Cannon GW, Teng C-C, Michaud K, Ibrahim S et al (2016) Changes in body mass related to the initiation of disease-modifying therapies in rheumatoid arthritis. Arthritis Rheumatol 68:1818–1827 26882094 10.1002/art.39647 PMC4963297

[25] Baker JF, Sauer B, Teng C-C, George M, Cannon GW, Ibrahim S et al (2018) Initiation of disease-modifying therapies in rheumatoid arthritis is associated with changes in blood pressure. JCR J Clin Rheumatol 24:203–209 29664818 10.1097/RHU.0000000000000736 PMC7461421

[26] Li N, Gou Z-P, Du S-Q, Zhu X-H, Lin H, Liang X-F et al (2022) Effect of JAK inhibitors on high- and low-density lipoprotein in patients with rheumatoid arthritis: a systematic review and network meta-analysis. Clin Rheumatol 41:677–688 34993729 10.1007/s10067-021-06003-z

[27] Ytterberg SR, Bhatt DL, Mikuls TR, Koch GG, Fleischmann R, Rivas JL et al (2022) Cardiovascular and cancer risk with tofacitinib in rheumatoid arthritis. N Engl J Med 386:316–326 35081280 10.1056/NEJMoa2109927

[28] Chaplin H, Sekhon M, Godfrey E The challenge of exercise (non-)adherence: a scoping review of methods and techniques applied to improve adherence to physical activity and exercise in people with inflammatory arthritis. Rheumatol Adv Pract [Internet]. 2023 [cited 2024 Sep 18];7. Available from: https://www.ncbi.nlm.nih.gov/pmc/articles/PMC9880978/10.1093/rap/rkac096 PMC9880978 36726735

[29] Kreps DJ, Halperin F, Desai SP, Zhang ZZ, Losina E, Olson AT et al (2018) Association of weight loss with improved disease activity in patients with rheumatoid arthritis: a retrospective analysis using electronic medical record data. Int J Clin Rheumatol 13:1–10 10.4172/1758-4272.1000154 PMC5875117 29606976

[30] Klingberg E, Björkman S, Eliasson B, Larsson I, Bilberg A (2020) Weight loss is associated with sustained improvement of disease activity and cardiovascular risk factors in patients with psoriatic arthritis and obesity: a prospective intervention study with two years of follow-up. Arthritis Res Ther 22:254 33092646 10.1186/s13075-020-02350-5 PMC7583178

